# Novel drug delivery system using acoustic control of intratumoral drug distribution

**DOI:** 10.1186/2050-5736-3-S1-P48

**Published:** 2015-06-30

**Authors:** Ken-ichi Kawabata, Maruoka Takashi, Rei Asami, Reiko Ashida

**Affiliations:** 1 Hitachi Ltd., Kokubunji, Japan; 2 Osaka Medical Center for Cancer and Cardiovascular Diseases, Osaka, Japan

## Background/introduction

An approach of drug delivery to hypovascular tumor tissues is proposed. For such tumor tissues, it is difficult to deliver anti-tumor agents at enough concentration and duration by either systemic or local administration. If we could manipulate the distribution of locally injected drugs by ultrasound, an efficient drug therapy would be possible. We have found that locally injected perfluorocarbon droplets (PCNDs) induce mechanical effects when exposed to pulsed HIFU at intensity of several kW/cm2 and further that controllable tissue destruction is possible with the effects. In this paper, preliminary results on a new drug delivery system (DDS) by utilizing such effects will be presented.

## Methods

All ultrasound exposures were performed using a focused ultrasound transducer with a diameter of 78 mm and F number of 1.0 at 1.1 MHz. Typically, ultrasound pulses with an intensity of 3 kW/cm2 (pulse duration: 300 cycles, PRF: 1 kHz, total exposure time: 60 s) was applied to samples or animals in a water tank filled with degassed water kept at 37 degree Celsius. Samples for *in vitro* experiments were polyacrylamide gels having closed liquid regions. In the regions, PCNDs and sham drug particles (black ink) were incorporated. Samples for *ex vivo* experiments were freshly excised chicken breast tissues. Before ultrasound application, PCNDs were injected. *In vivo* experiments were performed with CDF1 mice subcutaneously inoculated with Colon 26 tumor tissues. Before ultrasound application, PCNDs and/or an anti-tumor drug (Adriamycin) were injected into the tumor.

## Results and conclusions

1. *In vitro* experiments: It was found that the pulsed HIFU application induced the drug transportation toward the transducer. After a drug transportation, the drug-distributed region could be enlarged by shifting the ultrasound focus position. 2. *Ex vivo* experiments: When pulsed HIFU was applied to tissues samples in the absence of PCND, no damages were observed, while white opaque lesions were created when pulsed HIFU were applied to the PCND-injected regions. With microscopic observation, it was found that the tissue structures in the opaque regions were completely destroyed. By using a spatula, those regions were easily removed. 3. *In vivo* experiments: When pulsed HIFU were applied to tumor tissues without PCND injection, only short-term (2-3 days) delay in tumor growth was observed. At the same ultrasound conditions, the injection of PCND prior to the pulsed HIFU resulted in a significant brightness increase during application in diagnostic ultrasound scanner and further resulted in an enhanced tumor growth delay for about a week. When PCND and Adriamycin instead of PCND alone were injected to the tumor and the pulsed HIFU was then applied, a drastic tumor growth inhibition was observed. The tumor growth was completely prohibited for two weeks. In two of four cases, regrowth was observed after the two week observation but in the rest two tumor tissues did not regrowth in 30 days observation. No significant anti-tumor effects were observed when Adriamycin was injected and pulsed HIFU was not applied.

Our results demonstrated that the combination of a pulsed ultrasound and PCND can destroy tissue structures and further control the distribution of anti-tumor drugs. Such effect can suppress the growth of tumor tissues which cannot be achieved by the combination of ultrasound and PCND or anti-tumor drug alone.

